# Supplementary material for: Resolving the full spectrum of human genome variation using Linked-Reads
Source: Genome Res. 2019 Apr;29(4):635–45. doi: 10.1101/gr.234443.118 (PMC6442396; doi:10.1101/gr.234443.118)
Supplement: Supplemental Material [file supp_gr.234443.118_Supplemental_longranger-2.2.zip › longranger-2.2.tar.bz2!/lr2.2-public/lib/stan/cmdstan/stan/doc/api/html/math_2matrix_8hpp.html]

Stan: src/stan/math/matrix.hpp File Reference


|  |  |
| --- | --- |
|  | Stan  2.5.0  probability, sampling & optimization |


- Main Page
- Related Pages
- Namespaces
- Classes
- Files

- File List
- File Members

All Classes Namespaces Files Functions Variables Typedefs Enumerations Enumerator Friends Macros Pages

- src
- stan
- math

matrix.hpp File Reference

`#include <stan/math/matrix/Eigen.hpp>`  
`#include <stan/math/matrix/accumulator.hpp>`  
`#include <stan/math/matrix/add.hpp>`  
`#include <stan/math/matrix/append_row.hpp>`  
`#include <stan/math/matrix/array_builder.hpp>`  
`#include <stan/math/matrix/assign.hpp>`  
`#include <stan/math/matrix/block.hpp>`  
`#include <stan/math/matrix/append_col.hpp>`  
`#include <stan/math/matrix/cholesky_decompose.hpp>`  
`#include <stan/math/matrix/col.hpp>`  
`#include <stan/math/matrix/cols.hpp>`  
`#include <stan/math/matrix/columns_dot_product.hpp>`  
`#include <stan/math/matrix/columns_dot_self.hpp>`  
`#include <stan/math/matrix/common_type.hpp>`  
`#include <stan/math/matrix/containers_conversion.hpp>`  
`#include <stan/math/matrix/crossprod.hpp>`  
`#include <stan/math/matrix/cumulative_sum.hpp>`  
`#include <stan/math/matrix/determinant.hpp>`  
`#include <stan/math/matrix/diag_matrix.hpp>`  
`#include <stan/math/matrix/diag_post_multiply.hpp>`  
`#include <stan/math/matrix/diag_pre_multiply.hpp>`  
`#include <stan/math/matrix/diagonal.hpp>`  
`#include <stan/math/matrix/dims.hpp>`  
`#include <stan/math/matrix/distance.hpp>`  
`#include <stan/math/matrix/divide.hpp>`  
`#include <stan/math/matrix/dot_product.hpp>`  
`#include <stan/math/matrix/dot_self.hpp>`  
`#include <stan/math/matrix/eigenvalues_sym.hpp>`  
`#include <stan/math/matrix/eigenvectors_sym.hpp>`  
`#include <stan/math/matrix/elt_divide.hpp>`  
`#include <stan/math/matrix/elt_multiply.hpp>`  
`#include <stan/math/matrix/exp.hpp>`  
`#include <stan/math/matrix/fill.hpp>`  
`#include <stan/math/matrix/get_lp.hpp>`  
`#include <stan/math/matrix/get_base1.hpp>`  
`#include <stan/math/matrix/get_base1_lhs.hpp>`  
`#include <stan/math/matrix/head.hpp>`  
`#include <stan/math/matrix/initialize.hpp>`  
`#include <stan/math/matrix/inverse.hpp>`  
`#include <stan/math/matrix/inverse_spd.hpp>`  
`#include <stan/math/matrix/log.hpp>`  
`#include <stan/math/matrix/log_determinant.hpp>`  
`#include <stan/math/matrix/log_determinant_ldlt.hpp>`  
`#include <stan/math/matrix/log_determinant_spd.hpp>`  
`#include <stan/math/matrix/log_softmax.hpp>`  
`#include <stan/math/matrix/log_sum_exp.hpp>`  
`#include <stan/math/matrix/max.hpp>`  
`#include <stan/math/matrix/mdivide_left.hpp>`  
`#include <stan/math/matrix/mdivide_left_ldlt.hpp>`  
`#include <stan/math/matrix/mdivide_left_tri.hpp>`  
`#include <stan/math/matrix/mdivide_left_tri_low.hpp>`  
`#include <stan/math/matrix/mdivide_right.hpp>`  
`#include <stan/math/matrix/mdivide_right_ldlt.hpp>`  
`#include <stan/math/matrix/mdivide_right_tri.hpp>`  
`#include <stan/math/matrix/mdivide_right_tri_low.hpp>`  
`#include <stan/math/matrix/mean.hpp>`  
`#include <stan/math/matrix/meta.hpp>`  
`#include <stan/math/matrix/min.hpp>`  
`#include <stan/math/matrix/minus.hpp>`  
`#include <stan/math/matrix/multiply.hpp>`  
`#include <stan/math/matrix/multiply_lower_tri_self_transpose.hpp>`  
`#include <stan/math/matrix/num_elements.hpp>`  
`#include <stan/math/matrix/prod.hpp>`  
`#include <stan/math/matrix/promote_common.hpp>`  
`#include <stan/math/matrix/promoter.hpp>`  
`#include <stan/math/matrix/promote_scalar.hpp>`  
`#include <stan/math/matrix/promote_scalar_type.hpp>`  
`#include <stan/math/matrix/qr_Q.hpp>`  
`#include <stan/math/matrix/qr_R.hpp>`  
`#include <stan/math/matrix/quad_form.hpp>`  
`#include <stan/math/matrix/quad_form_diag.hpp>`  
`#include <stan/math/matrix/rank.hpp>`  
`#include <stan/math/matrix/resize.hpp>`  
`#include <stan/math/matrix/row.hpp>`  
`#include <stan/math/matrix/rows.hpp>`  
`#include <stan/math/matrix/rows_dot_product.hpp>`  
`#include <stan/math/matrix/rows_dot_self.hpp>`  
`#include <stan/math/matrix/sd.hpp>`  
`#include <stan/math/matrix/segment.hpp>`  
`#include <stan/math/matrix/singular_values.hpp>`  
`#include <stan/math/matrix/size.hpp>`  
`#include <stan/math/matrix/softmax.hpp>`  
`#include <stan/math/matrix/sort.hpp>`  
`#include <stan/math/matrix/sort_indices.hpp>`  
`#include <stan/math/matrix/squared_distance.hpp>`  
`#include <stan/math/matrix/stan_print.hpp>`  
`#include <stan/math/matrix/sub_col.hpp>`  
`#include <stan/math/matrix/sub_row.hpp>`  
`#include <stan/math/matrix/subtract.hpp>`  
`#include <stan/math/matrix/sum.hpp>`  
`#include <stan/math/matrix/tail.hpp>`  
`#include <stan/math/matrix/tcrossprod.hpp>`  
`#include <stan/math/matrix/trace.hpp>`  
`#include <stan/math/matrix/trace_gen_inv_quad_form_ldlt.hpp>`  
`#include <stan/math/matrix/trace_gen_quad_form.hpp>`  
`#include <stan/math/matrix/trace_inv_quad_form_ldlt.hpp>`  
`#include <stan/math/matrix/trace_quad_form.hpp>`  
`#include <stan/math/matrix/transpose.hpp>`  
`#include <stan/math/matrix/typedefs.hpp>`  
`#include <stan/math/matrix/variance.hpp>`

Go to the source code of this file.


---

[ Stan Home Page ]

*© 2011–2014,
Stan Development Team.*
